# The application of a workflow integrating the variable reproducibility and harmonizability of radiomic features on a phantom dataset

**DOI:** 10.1371/journal.pone.0251147

**Published:** 2021-05-07

**Authors:** Abdalla Ibrahim, Turkey Refaee, Ralph T. H. Leijenaar, Sergey Primakov, Roland Hustinx, Felix M. Mottaghy, Henry C. Woodruff, Andrew D. A. Maidment, Philippe Lambin

**Affiliations:** 1 The D-Lab, Department of Precision Medicine, GROW- School for Oncology, Maastricht University, Maastricht, The Netherlands; 2 Department of Radiology and Nuclear Medicine, Maastricht University Medical Centre+, Maastricht, The Netherlands; 3 Division of Nuclear Medicine and Oncological Imaging, Department of Medical Physics, University Hospital of Liège and GIGA CRC-in vivo imaging, University of Liège, Liege, Belgium; 4 Department of Nuclear Medicine and Comprehensive Diagnostic Centre Aachen (CDCA), University Hospital RWTH Aachen University, Aachen, Germany; 5 Faculty of Applied Medical Sciences, Department of Diagnostic Radiology, Jazan University, Jazan, Saudi Arabia; 6 Oncoradiomics SA, Liege, Belgium; 7 Department of Radiology, Perelman School of Medicine, University of Pennsylvania, Philadelphia, PA, United States of America; Washington University in St. Louis, UNITED STATES

## Abstract

Radiomics–the high throughput extraction of quantitative features from medical images and their correlation with clinical and biological endpoints- is the subject of active and extensive research. Although the field shows promise, the generalizability of radiomic signatures is affected significantly by differences in scan acquisition and reconstruction settings. Previous studies reported on the sensitivity of radiomic features (RFs) to test-retest variability, inter-observer segmentation variability, and intra-scanner variability. A framework involving robust radiomics analysis and the application of a post-reconstruction feature harmonization method using ComBat was recently proposed to address these challenges. In this study, we investigated the reproducibility of RFs across different scanners and scanning parameters using this framework. We analysed thirteen scans of a ten-layer phantom that were acquired differently. Each layer was subdivided into sixteen regions of interest (ROIs), and the scans were compared in a pairwise manner, resulting in seventy-eight different scenarios. Ninety-one RFs were extracted from each ROI. As hypothesized, we demonstrate that the reproducibility of a given RF is not a constant but is dependent on the heterogeneity found in the data under analysis. The number (%) of reproducible RFs varied across the pairwise scenarios investigated, having a wide range between 8 (8.8%) and 78 (85.7%) RFs. Furthermore, in contrast to what has been previously reported, and as hypothesized in the robust radiomics analysis framework, our results demonstrate that ComBat cannot be applied to all RFs but rather on a percentage of those–the “ComBatable” RFs–which differed depending on the data being harmonized. The number (%) of reproducible RFs following ComBat harmonization varied across the pairwise scenarios investigated, ranging from 14 (15.4%) to 80 (87.9%) RFs, and was found to depend on the heterogeneity in the data. We conclude that the standardization of image acquisition protocols remains the cornerstone for improving the reproducibility of RFs, and the generalizability of the signatures developed. Our proposed approach helps identify the reproducible RFs across different datasets.

## Introduction

With the advancement and involvement of artificial intelligence in performing high-level tasks, its application has been extensively researched in the field of medical imaging analysis [1]. Radiomics–the high throughput extraction of quantitative features from medical imaging to find correlations with biological or clinical outcomes [2–4]–is currently one of the most commonly used quantitative imaging analysis methods in medical imaging.

A major area of research in the field of radiomics is the selection of robust and informative image features to be used as input for machine learning models [5]. Evidence suggests that radiomic features (RFs) are sensitive to differences in several factors, including make and type of imaging scanner, reconstruction settings, and protocols used to acquire the images [6,7]. Studies on the reproducibility of RFs across test-retest [8,9]; or across scans of a phantom made on the same scanner using different exposure levels, while fixing other parameters [10]; or across scans of a phantom using different acquisition and reconstruction parameters [11] highlighted the high sensitivity of RFs to variations within datasets.

The above-mentioned studies focused on the reproducibility of RFs in limited settings, such as test-retest, inter-observer variability, and intra-scanner variability. As these studies reported significant differences in groups of RFs, it is only intuitive that adding more variation to image acquisition and reconstruction will further dampen the reproducibility of RFs. These findings indicate that ignoring data heterogeneity will influence the performance and generalizability of the models developed, especially in studies where training and validation sets are independent. Therefore, a global initiative–the Image Biomarkers Standardization Initiative (IBSI)–has been initiated in an effort to standardize the extraction of image biomarkers (RFs) from medical images [12]. The IBSI aims to standardize both the computation of RFs and the image processing steps required before RF extraction. However, little attention has been paid in the bulk of literature to date to the heterogeneity in image acquisition and reconstruction when performing radiomics analysis. As the goal of radiomics research is to employ quantitative imaging features as clinical biomarker, the issue of accurate measurement and reproducibility must be addressed [13]. Biomarkers are defined as “the objective indications of medical state observed from outside the patient–which can be measured reproducibly”. Therefore, reproducible measurement is a corner stone in choosing a biomarker. In essence, RFs that cannot be reproduced cannot be compared or selected as biomarkers.

Combining Batches (ComBat) harmonization is a method that was introduced for removing the effects of machinery and protocols used to extract gene expression data, in order to make gene expression data acquired at different centres comparable [14]. ComBat is a method that performs location and scale adjustments of the values presented to remove the discrepancies in RF values introduced by technical differences in the images. These sources of variation are further referred to as batch effects. ComBat was subsequently adopted in radiomics analysis, and some studies reported that ComBat outperforms other harmonization methods (e.g, histogram-matching, voxel size normalization, and singular value decomposition) in radiomics analyses [15,16]. Several radiomics studies have reported on the successful application of ComBat in removing the differences in RFs introduced by different vendors and acquisition protocols [17–21]. These studies investigated the differences in radiomic RF distributions across different batches following the application of ComBat harmonization. In contrast to gene expression arrays, RFs have different definitions, and the batch effect might vary for each RF. Using phantom data allows one to study the variations in a given RF extracted from scans acquired with different scanners/reconstruction settings and to attribute these variations to the changes in acquisition and reconstruction, which in theory ComBat harmonization is designed to mitigate. However, we are not aware of any study that has performed a systematic evaluation of the performance of ComBat harmonization across variations between imaging parameters, which is the one of the objectives of this study.

**Fig 1:**
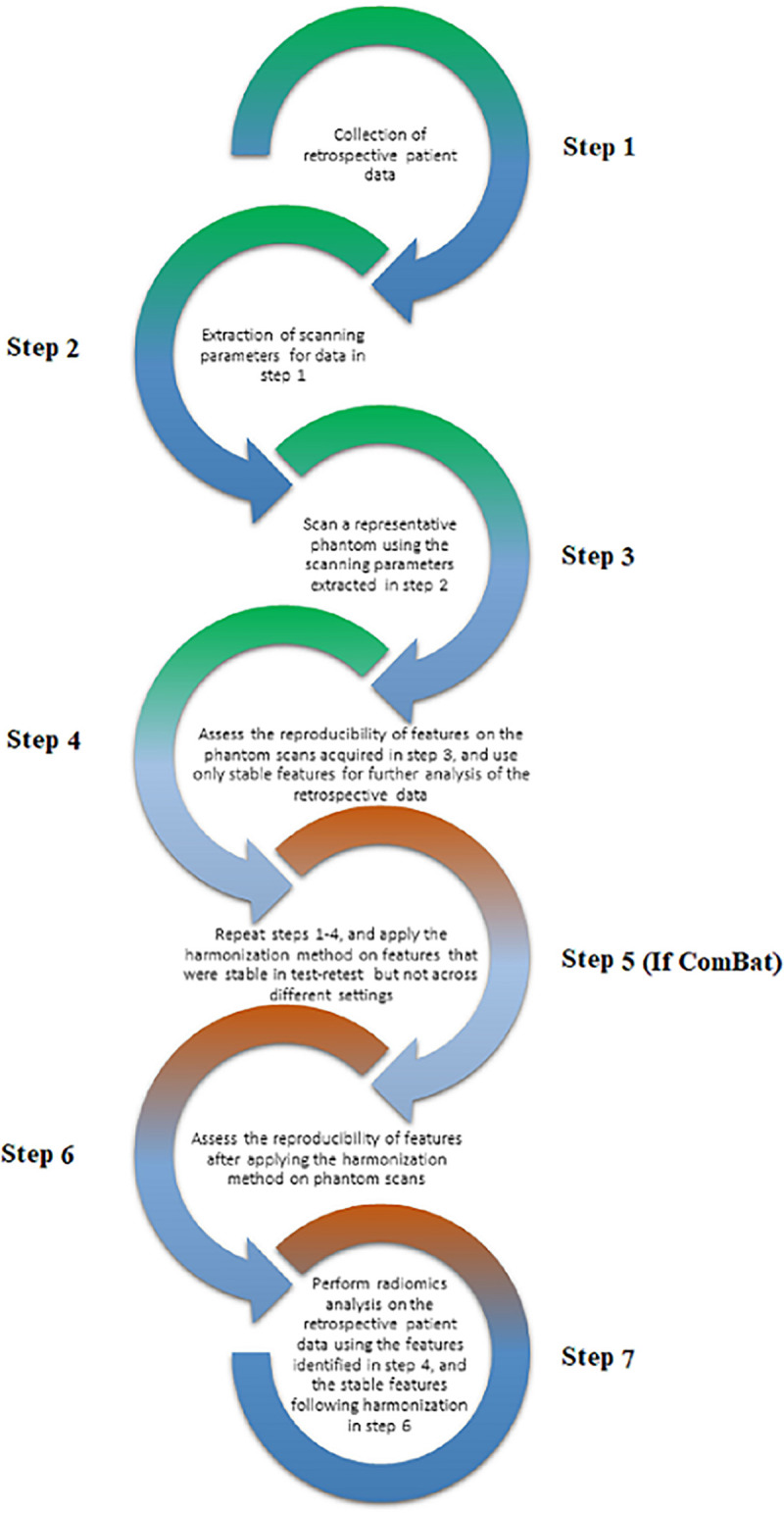
The proposed framework (reprinted with permission from [22]).

Ibrahim et al. (2020) [22] have proposed a new radiomics workflow (Fig 1) that tries to address the challenges current radiomics analyses face. The framework was proposed based on mathematical considerations of the complexity of medical imaging, and RFs’ mathematical definitions. Our framework is based on the hypothesis that the reproducibility of a given RF is a not constant, but depends on the variations of image acquisition and reconstruction in the data under study. Furthermore, for ComBat to be applicable in radiomics, radiomic RF values for a given region of interest obtained after ComBat must be (nearly) identical, regardless of differences in acquisition and reconstruction.

Our general objective is to set-up the requirements for selecting biomarkers from RFs, to ease their incorporation into clinical decision support systems. We hypothesize that variations in image acquisition and reconstruction will variably affect RFs reproducibility. Furthermore, the performance of ComBat on a given RF is dependent on those variations, i.e, a given RF can be successfully harmonized with ComBat with specific variations in the imaging parameters but not others. We investigate these hypotheses on CT scans using a ten-layer radiomics phantom, which was scanned with different acquisition and reconstruction parameters on various scanner models.

## Methods

### Phantom data

The publicly available Credence Cartridge Radiomics (CCR) phantom data, found in The Cancer Imaging Archive (TCIA.org) [23,24], was used. The CCR phantom is composed of 10 different layers that correspond to different texture patterns spanning a range of −900 to +700 Hounsfield units (HU). Each layer of the phantom was further subdivided into 16 distinct regions of interest (ROI) with cubic volume of 8 cm^3^, resulting in a total of 2080 ROIs available for further analysis. The phantom was originally scanned using 17 different imaging protocols from four medical institutes using equipment from different vendors and a variety of acquisition and reconstruction parameters. Four of the scans lacked ROI definitions, thus to maintain consistency, these were not included. The remaining 13 scans are as follows: seven different scans acquired on GE scanners, five different scans acquired on Philips scanners, and one scan acquired on a Siemens scanner (Tables 1 and 2).

**Table 1:** CT acquisition parameters*.

Scan	Vendor	Model	Scan Options	Effective mAs**	kVp
CCR1-001	GE	Discovery CT750 HD	HELICAL	81	120
CCR1-002	GE	Discovery CT750 HD	AXIAL	300	120
CCR1-003	GE	Discovery CT750 HD	HELICAL	122	120
CCR1-004	GE	Discovery ST	HELICAL	143	120
CCR1-005	GE	LightSpeed RT	HELICAL	1102	120
CCR1-006	GE	LightSpeed RT16	HELICAL	367	120
CCR1-007	GE	LightSpeed VCT	HELICAL	82	120
CCR1-008	Philips	Brilliance Big Bore	HELICAL	320	120
CCR1-009	Philips	Brilliance Big Bore	HELICAL	369	120
CCR1-010	Philips	Brilliance Big Bore	HELICAL	320	120
CCR1-011	Philips	Brilliance Big Bore	HELICAL	369	120
CCR1-012	Philips	Brilliance 64	HELICAL	372	120
CCR1-013	SIEMENS	Sensation Open	AXIAL	26–70	120

* Values are directly extracted from the publicly available imaging tags.

**Table 2:** CT reconstruction parameters*.

Scan	Convolution Kernel	Filter Type	Slice thickness (mm)	Pixel spacing (mm)
CCR1-001	STANDARD	BODY FILTER	2.5	0.49
CCR1-002	STANDARD	BODY FILTER	2.5	0.70
CCR1-003	STANDARD	BODY FILTER	2.5	0.78
CCR1-004	STANDARD	BODY FILTER	2.5	0.98
CCR1-005	STANDARD	BODY FILTER	2.5	0.98
CCR1-006	STANDARD	BODY FILTER	2.5	0.98
CCR1-007	STANDARD	BODY FILTER	2.5	0.74
CCR1-008	B	B	3	0.98
CCR1-009	C	C	3	0.98
CCR1-010	B	B	3	1.04
CCR1-011	B	B	3	1.04
CCR1-012	B	B	3	0.98
CCR1-013	B31s	0	3	0.54

* Values are directly extracted from the publicly available imaging tags.

### Radiomic features extraction

For each ROI, quantitative imaging features were calculated using the open source Pyradiomics (V 2.0.2). The software contains IBSI-compliant RFs, with deviations highlighted in the feature definitions. For the extraction step, no changes to the original slice thickness or pixel spacing of the scans were applied. To reduce noise and computational requirements, images were pre-processed by binning voxel greyscale values into bins with a fixed width of 25 HUs prior to extracting RFs. The extracted features included HU intensity features, shape features, and texture features describing the spatial distribution of voxel intensities using 5 texture matrices (i.e., grey-level co-occurrence (GLCM), grey-level run-length (GLRLM), grey-level size-zone (GLSZM), grey-level dependence (GLDM), and neighbourhood grey-tone difference matrix (NGTDM)). Detailed description of the features can be found online at https://pyradiomics.readthedocs.io/en/latest/features.html.

### ComBat harmonization

ComBat employs empirical Bayes methods to estimate the differences in feature values attributed to a batch effect. Empirical Bayes methods are able to estimate the prior distribution from a given dataset via statistical inference. In the context of radiomics, ComBat assumes that feature values can be approximated by the equation:

$$Y_{ij}=\alpha +\beta X_{ij}+\gamma_{i}+\delta_{i}\varepsilon_{ij}$$ (1)

where α is the average value for feature Y_ij_ for ROI j on scanner i; X is a design matrix of the covariates of interest; β is the vector of regression coefficients corresponding to each covariate; γ_i_ is the additive effect of scanner i on features, which is presupposed to follow a normal distribution; δ_i_ is the multiplicative scanner effect, which is presupposed to follow an inverse gamma-distribution; and ε_ij_ is an error term, presupposed to be normally distributed with zero mean [17]. ComBat performs feature transformation based on the empirical Bayes prior estimates for γ and δ for each batch:

$$Y_{ij}^{ComBat}=\frac{\left(Y_{ij}-\hat{\alpha }-\hat{\beta }X_{ij}-\gamma_{i}^{*}\right)}{\delta_{i}^{*}}+\hat{a}+\hat{\beta }X_{ij}$$ (2)

where $\hat{\alpha }$ and $\hat{\beta }$ are estimators of parameters α and β, respectively. $\gamma_{i}^{*}$ and $\delta_{i}^{*}$ are the empirical Bayes estimates of γ_i_ and δ_i_, respectively [17].

### Statistical analysis

To assess the agreement of a given RF for the same ROI scanned using different settings and scanners, the concordance correlation coefficient (CCC) was calculated using epiR (version 0.9–99) [25] on R [26] (version 3.5.1), using R studio (version 1.1.456) [27]. The CCC is used to evaluate the agreement between paired readings [28], and provides the measure of concordance as a value between 1 and -1, where 0 represents no concordance, 1 represents a perfect direct positive concordance, and -1 indicates a perfect inverse concordance. It further takes into account the rank and value of the RFs.

The analysis of the reproducibility before and after ComBat harmonization was performed in a pairwise manner, resulting in 78 different investigated scenarios. To assess differences in RF stability for differing data, the reproducibility of radiomics RFs across scans within a wide spectrum of scenarios was calculated. Data ranging from differences in a single acquisition or reconstruction parameter, to scans acquired using entirely different settings (See S1 Table) were included. To identify reproducible radiomics, the CCC was calculated for all RFs for all ROIs across the 78 investigated scenarios. A cut-off of CCC>0.9, as found in the literature, suggests that a value < 0.9 indicates poor concordance [29].To identify the RFs that could be harmonized using ComBat, the pairwise CCC was calculated following ComBat in each of the investigated 78 scenarios. We applied ComBat using R package “SVA” (version 3.30.1) [30]. As the RFs are calculated for the same ROI but for different scans, the agreement in RF value is expected to be high following ComBat harmonization. Thus, RFs that had a CCC<0.9 were considered to be not harmonizable with ComBat. The extracted RFs and code used in this work is publicly available on https://github.com/AbdallaIbrahim/The-reproducibility-and-ComBatability-of-Radiomic-features.

## Results

### Reproducible radiomic features

For each ROI, a total of 91 RFs were extracted. The number (percentage) of reproducible RFs in each pairwise comparison ranged from 9 (8.8%) to 78 (85.7%) RFs, depending on the variations in acquisition and reconstruction of the scans (Table 3). The highest concordance in feature values (85.7%) was observed between the two Philips scans (CCR1-010 and CCR1-011) that were acquired using the same scanner model, and the same acquisition and reconstruction parameters except for the effective mAs, which differed by just 15% (Tables 1 and 2).

**Table 3:** The number (percentage) of concordant RFs before ComBat harmonization between pairwise combinations of scans with different acquisition and reconstruction.

	CCR1-001	CCR1-002	CCR1-003	CCR1-004	CCR1-005	CCR1-006	CCR1-007	CCR1-008	CCR1-009	CCR1-010	CCR1-011	CCR1-012
**CCR1-002**	38 (41.76%)											
**CCR1-003**	46 (50.55%)	59 (64.84%)										
**CCR1-004**	18 (19.78%)	34 (37.36%)	25 (27.47%)									
**CCR1-005**	13 (14.29%)	23 (25.27%)	17 (18.68%)	66 (72.53%)								
**CCR1-006**	16 (17.58%)	24 (26.37%)	18 (19.78%)	71 (78.02%)	69 (75.82%)							
**CCR1-007**	49 (53.85%)	65 (71.43%)	67 (73.63%)	21 (23.08%)	14 (15.38%)	14 (15.38%)						
**CCR1-008**	8 (8.79%)	12 (13.19%)	14 (15.38%)	41 (45.05%)	34 (37.36%)	47 (51.65%)	10 (10.99%)					
**CCR1-009**	9 (9.89%)	19 (20.88%)	13 (14.29%)	67 (73.63%)	65 (71.43%)	74 (81.32%)	11 (12.09%)	48 (52.75%)				
**CCR1-010**	8 (8.79%)	10 (10.99%)	13 (14.29%)	32 (35.16%)	21 (23.08%)	27 (29.67%)	11 (12.09%)	59 (64.84%)	34 (37.36%)			
**CCR1-011**	8 (8.79%)	11 (12.09%)	12 (13.19%)	45 (49.45%)	34 (37.36%)	42 (46.15%)	11 (12.09%)	57 (62.64%)	52 (57.14%)	78 (85.71%)		
**CCR1-012**	8 (8.79%)	13 (14.29%)	12 (13.19%)	21 (23.08%)	16 (17.58%)	22 (24.18%)	10 (10.99%)	61 (67.03%)	36 (39.56%)	71 (78.02%)	69 (75.82%)	
**CCR1-013**	51 (56.04%)	44 (48.35%)	47 (51.65%)	41 (45.05%)	34 (37.36%)	32 (35.16%)	48 (52.75%)	12 (13.19%)	23 (25.27%)	10 (10.99%)	9 (9.89%)	10 (10.99%)

The more profound the variations in scan acquisition parameters, the smaller the concordance of the extracted RFs (Tables 1–3 and S1).

As stated, in the best scenario (CCR1-010 and CCR1-011), 78 (85.7%) RFs were found to be reproducible, while 13 (14.3%) RFs were found not to be reproducible. Some RFs (n = 8) were found to be concordant across all pairs. These RFs were histogram-based RFs that take into account the value of a single pixel/voxel, without looking at the relationship between neighbouring pixels/voxels. These RFs are (i) original first order 10Percentile; (ii) original first order 90Percentile; (iii) original first order Maximum; (iv) original first order Mean (v) original first order Median; (vi) original first order Minimum; (vii) original first order Root Mean Squared; and (viii) original first order Total Energy. Nevertheless, the remainder (majority) of the RFs (including 10 histogram-based RFs) were not found to be reproducible across all pairs.

Looking at (Tables 1–3 and S1), we can consider subgroups of scans. Scans CCR1-001-007 were all acquired using the same imaging vendor (GE), but different scanner models and scanning parameters. The highest number of concordant RFs in this group was found between CCR1-004 and CCR1-006 (71 RFs), which were acquired on two different scanner models, but were scanned with identical scanning parameters except for the mAs. The lowest number of concordant RFs in this group was found between scans CCR1-001 and CCR1-005 (13 RFs), which were acquired on two different scanner models, with the same scanning parameters except for the pixel spacing and mAs. Scans CCR1-007 to CCR1-012 were all acquired using one of two Philips imaging vendors. The highest number of concordant RFs is documented above. The lowest number of concordant RFs was found between CCR1-009 and CCR-010 (34 RFs), which differed in terms of the mAs, convolution kernel, filter type and pixel spacing. Looking at the group of scans that were reconstructed to the same pixel spacing (CCR1-004 to CCR1-006, CCR1-008, CCR1-009, and CCR-012), the highest number of concordant RFs was observed between CCR1-006 and CCR1-009 (74 RFs), which were acquired using two different imaging vendors, but using similar acquisition and reconstruction parameters except for the slice thickness, and kernel. The lowest number of concordant RFs was found between CCR1-005 and CCR1-012 (16 RFs), which were acquired using different imaging vendors, and different acquisition and reconstruction parameters except for the kVp. Finally, comparing scans acquired with different vendors resulted in a lower number of concordant RFs compared to scans acquired with the scanners from the same imaging vendor, except for the scenario when the majority of acquisition and reconstruction parameters were mostly identical (CCR1-006 vs CCR1-009).

### ComBat harmonization

As previously shown in the literature, we used each scan as a different batch in the ComBat equation. ComBat was applied pairwise (78 different pairs) and the concordance between RFs was measured for each pair (Table 4). The percentage of RFs that became concordant following ComBat application ranged from 1.4% (71 concordant RFs increased to 72) to 344% (9 concordant RFs increased to 40).

**Table 4:** The number (percentage) of concordant RFs after ComBat harmonization between pairwise combinations of scans with different acquisition and reconstruction.

	CCR1-001	CCR1-002	CCR1-003	CCR1-004	CCR1-005	CCR1-006	CCR1-007	CCR1-008	CCR1-009	CCR1-010	CCR1-011	CCR1-012
**CCR1-002**	63 (69.23%)											
**CCR1-003**	69 (75.82%)	75 (82.42%)										
**CCR1-004**	48 (52.75%)	72 (79.12%)	57 (62.64%)									
**CCR1-005**	43 (47.25%)	60 (65.93%)	54 (59.34%)	72 (79.12%)								
**CCR1-006**	50 (54.95%)	63 (69.23%)	59 (64.84%)	76 (83.52%)	72 (79.12%)							
**CCR1-007**	70 (76.92%)	69 (75.82%)	74 (81.32%)	56 (61.54%)	49 (53.85%)	57 (62.64%)						
**CCR1-008**	27 (29.67%)	36 (39.56%)	36 (39.56%)	61 (67.03%)	54 (59.34%)	56 (61.54%)	28 (30.77%)					
**CCR1-009**	40 (43.96%)	57 (62.64%)	53 (58.24%)	76 (83.52%)	74 (81.32%)	81 (89.01%)	52 (57.14%)	57 (62.64%)				
**CCR1-010**	18 (19.78%)	22 (24.18%)	19 (20.88%)	54 (59.34%)	48 (52.75%)	48 (52.75%)	17 (18.68%)	68 (74.73%)	53 (58.24%)			
**CCR1-011**	14 (15.38%)	23 (25.27%)	25 (27.47%)	67 (73.63%)	59 (64.84%)	59 (64.84%)	16 (17.58%)	65 (71.43%)	67 (73.63%)	80 (87.91%)		
**CCR1-012**	16 (17.58%)	29 (31.87%)	28 (30.77%)	56 (61.54%)	48 (52.75%)	49 (53.85%)	16 (17.58%)	70 (76.92%)	53 (58.24%)	72 (79.12%)	74 (81.32%)	
**CCR1-013**	65 (71.43%)	75 (82.42%)	69 (75.82%)	65 (71.43%)	55 (60.44%)	59 (64.84%)	67 (73.63%)	35 (38.46%)	58 (63.74%)	35 (38.46%)	36 (39.56%)	34 (37.36%)

The highest number of concordant RFs following ComBat application was 80 (87.9%) RFs. In this scenario, a single acquisition parameter differed between the two scans (Philips, CCR1-010 and CCR1-011). ComBat application improved the concordance of only two RFs (80 RFs after ComBat compared to 78 RFs before), and failed to improve the concordance of the remaining 11 RFs. On the other hand, in cases where the differences in acquisition and reconstruction parameters differed more (e.g., CCR1-001 (GE) vs CCR1-007 (Philips)), the application of ComBat improved the concordance of 31 RFs, resulting in a total of 40 concordant RFs (~44% of the total number of RFs), more than 3 times the number of concordant RFs before harmonization. Furthermore, the successful application of ComBat on RFs depended on the variations in the batches defined. Only two RFs were found to be concordant in all pairwise scenarios following ComBat harmonization: (i) original first order Energy; and (ii) original gldm Small Dependence High Gray Level Emphasis; in addition to the 8 RFs mentioned above.

## Discussion

In this work, for our first objective to investigate RFs reproducibility, we show that the majority of RFs are affected to different amounts depending upon the variations in acquisition and reconstruction parameters. We also show that the reproducibility of a given RF is not constant, but rather it is dependent on the variations in the data under study, as seen in Table 3. We identified a number of RFs that were robust to the variations in scan acquisition in the dataset we analysed. These RFs could be used without any post–processing harmonization. While the same dataset has been analysed for similar purposes previously [11,21], we analysed the data differently, and report different results than those studies. Our results show a substantial intra-scanner variability, and even greater inter-scanner variability, which is in line with other previous findings [10,31,32]. Only eight RFs (~9%) of the extracted RFs showed insensitivity to the differences in acquisition shown in Tables 1 and 2, and could be directly used to build radiomic signatures. The rest of the RFs (91%) could not be used without addressing the acquisition differences. Our sub-groups analysis showed that changes in pixel spacing and convolution kernel have more profound effects on the reproducibility of RFs, compared to variations limited solely to the effective mAs, scanner model or imaging vendor used. While the percentages reported are representative of the reproducibility of RFs in the data analysed, it highlights the sensitive nature of RFs, and helps set guidelines to preselect meaningful and reproducible RFs. We deduce that the use of RFs extracted from scans acquired with different hardware and parameters, without addressing the issue of reproducibility and harmonization, can lead to spurious results as the vast majority of RFs are sensitive to even minor variations in image acquisition and reconstruction. Therefore, models developed using RFs with large unexplained variances will most likely not be generalizable.

As our second aim, we investigated the applicability of ComBat harmonization to removing differences in RF values attributed to batch effects. Studies [11,21] have reported on the reproducibility of RFs on the same or a similar dataset to the one we analysed. However, our findings and conclusions vary significantly from theirs. In contrast to previous studies, we are the first to report that the reproducibility of RFs is dependent on the variations in the data under analysis. Previous studies referred to RFs as generally reproducible or non-reproducible. Our analysis shows that a given RF can be reproducible in some scenarios and not in the others, depending on the variations in acquisition and reconstruction parameters. Moreover, ComBat was mathematically defined to remove one (technical) batch effect at a time while considering all the biologic covariates at the same time. However, as our results show (Tables 3 and 4), the variations in acquisition and reconstruction parameters within one scanner, at least in some instances, have a stronger impact on the reproducibility of RFs than the variations between two scanners. As such, grouping the scans by the scanner type is not generally the way to define “batches” in the ComBat equation [14]. In contrast to what is reported in the literature, our analysis shows ComBat did not perform uniformly on most of the RFs when there were variations in the batches being harmonized. In contrast to those studies, we employed the concordance correlation coefficient (CCC) to assess the reproducibility of RFs, since the aim of harmonization is to improve the reproducibility of data. We did not use the increment of model performance as a measure for the success of harmonization for several reasons. First, the aim of harmonization is to improve the reproducibility of RFs, and ultimately the generalizability of the developed signatures, and not their model performance [33]. Second, by assuming that an increment in the model performance following harmonization is an indication that the harmonization is successful carries with it the assumption that radiomic models decode the information under analysis; this is against the essence of the study, which is to investigate whether radiomics has that potential or not. However, by using the CCC, we ensure that the results generated are based on reproducible RFs, and are therefore generalizable, regardless of the change in model performance. Furthermore, the aim of ComBat harmonization is only to remove the variance in RF values attributed to the batch effects, while maintaining the biologic information. As such, using ComBat to correct batch effects directly on patient data without providing the correct biological covariates that actually do have an effect on RF values will lead to loss of biological signals. This is because ComBat tries to harmonize the distribution of the RF across different batches, and without providing the correct biological covariates that have effects on RF values, ComBat assumes that the variations in RF value are only attributed to the defined batch, and thus would not perform uniformly as shown in Table 3. In clinical settings, this is by default spurious, as the differences in RF values are attributed to both the machine and the biology/physiology. As the aim of radiomics studies is to investigate the biological correlations of RFs, we are unable to actually provide a list of biologic covariates that influence the values. In addition, each time an observation is added to the data being harmonized, ComBat has to be re-performed, and models have to be refitted, as the estimated batch effects will change each time. Therefore, the harmonization of patient RFs should follow the process of estimating fixed batch effects on phantom data, then applying the location/scale shift estimated from the phantom data on patient data, as previously described by Ibrahim et al [22].

The pairwise approach we used shows how the variations in scan acquisition and reconstruction parameters affect the reproducibility of RFs. Therefore, aside from probably a few RFs, the reproducibility of the majority of the RFs cannot be guessed in untested scenarios. The workflow (Fig 1) addresses this problem by introducing the assessment of RF reproducibility on representative phantom data. This workflow differs from existing radiomics workflows by the addition of an intermediary RF pre-selection step between RF extraction and RF selection by one of two approaches: (i) only extracting the reproducible RFs for analysis; (ii) extracting and harmonizing the ‘ComBatable’ RFs before RF selection and model building. The application of ComBat and the definition of what constitutes a ‘batch’ should be performed based on the data being analysed, as could be deduced from Tables 3 and 4. For example, RFs extracted from scans acquired with different scanner models, but similar settings were found to be more concordant than RFs extracted with the same scanner model but with profound differences in acquisition and reconstruction parameters. Our proposed radiomics analysis workflow would ensure that the RFs being analysed are not affected by scan acquisition differences, and henceforth, signatures built would be more robust and generalizable. The first part of the model (steps 1–4), where only reproducible RFs are extracted and further analysed, might significantly limit the number of RFs used for further modelling. However, using the whole framework may significantly increase the number of RFs that can be used, depending on the data under study.

While the data used for this analysis are not representative of diagnostic clinical protocols and do not provide all technical details needed for proper analysis, our aim was to show that changes in scan acquisition and reconstruction parameters differently affect the majority of RFs. The variations in the reproducibility of RFs–as well as ComBat applicability–due to the heterogeneity in acquisition and reconstruction highlight the necessity of the standardization of image acquisition and reconstruction across centres. RFs have already been reported to be sensitive to test-retest [8,34], which is the acquisition of two separate scans using the same parameters, as well as to the variations in the parameters within the same scanner [10]. Adding the variable sensitivity of RFs to different acquisition and reconstruction parameters significantly lowers the number of RFs that could be used for the analysis of heterogeneous data. As there is currently a pressing desire to analyse big data, a sound methodology is needed to address the heterogeneity introduced by machinery in retrospective data. Nevertheless, we strongly recommend the start of imaging protocol standardization across centres to facilitate future quantitative imaging analysis.

Recently, there has been an attempt to modify ComBat methodology in radiomics analysis [35]. The authors added a modification to ComBat (B-ComBat), which adds Bootstrapping and Monte Carlo to the original ComBat. The other functionality of ComBat the authors investigated was to use one of the batches as a reference (M-ComBat). The authors compared the performance of the four versions of ComBat by comparing the performance of radiomic models developed after the use of each method. The authors reported that all the methods are equally effective [35]. Therefore, we anticipate that the modified ComBat functions will have the same limitations of the original ComBat we discussed above.

Another method to harmonize RFs that is currently gaining momentum is deep learning based harmonization. A recent study developed deep learning algorithms, which were reported to improve the reproducibility of RFs across variations in scanner type, acquisition protocols and reconstruction algorithms [36]. A more recent study [37] applied a similar approach to reduce the sensitivity of RFs to scanner types. The authors reported a significant improvement in the performance of radiomic models following harmonization. These studies highlight the potential efficacy of deep learning based harmonization methods.

One limitation of our study is in considering each scan as a separate batch effect (due to lack of data) while differences between pair batches are not similar (different numbers of varying parameters), which may have affected the performance of ComBat. Acquisition and reconstruction settings include a set of different parameters, which can singularly or collectively result in differences in RFs values. Another limitation is the lack of scans generated by other commonly used scanners and protocols in the clinics; and the lack of scans with the same settings acquired using different scanners, as the data currently available is limited to the changes introduced in the imaging parameters on the available scanners. While we did not investigate the added value of this approach on a clinical dataset, our focus in this study was in designing a framework to assess the reproducibility and ‘ComBatability’ of RFs. However, it is fair to assume that if RFs are not reproducible on phantom data, they would be equally, or possibly even more, unstable on patient datasets. For example, clinical data will be acquired at a variety of mAs values across a population of patients. Lastly, while Combat has been reported to outperform other harmonization methods in terms of apparent model performance, the systemic evaluation of the effects of these methods on the reproducibility of RFs, and the comparison with the effects of ComBat harmonization will be the aim of future studies, in addition to addressing the above mentioned limitations.

## Conclusion

In conclusion, we demonstrate that the reproducibility of RFs is not a constant, but changes with variations in the data acquisition and reconstruction parameters. Moreover, ComBat cannot be successfully applied on all RFs, and its successful application on a given RF is dependent on the heterogeneity of the dataset. We conclude that ComBat harmonization should not be blindly performed on patient data, but following the estimation of adjustment parameters on a phantom dataset. We anticipate that radiomics studies will benefit from our proposed harmonization workflow, as it allows comparison of a greater number of RFs, and enhances the generalizability of radiomic models. Yet, standardization of imaging protocols remains the cornerstone for improving the generalizability of prospective quantitative image studies. We recommend the standardization of scan acquisition across centres, especially in prospective clinical trials that include medical imaging; and/or the development of a specific imaging protocols for scans acquired to be used for quantitative imaging analysis.

## Supporting information

S1 Table The agreements and disagreements in the scanner models and scanning parameters in the pairwise comparisons. (DOCX)
