# Supplementary material for: The application of a workflow integrating the variable reproducibility and harmonizability of radiomic features on a phantom dataset
Source: PLoS One. 2021 May 7;16(5):e0251147. doi: 10.1371/journal.pone.0251147 (PMC8104396; doi:10.1371/journal.pone.0251147)
Supplement: S1 Table — (DOCX) [file pone.0251147.s001.docx]

| **S1 Table. The agreements and disagreements in the scanner models and scanning parameters in the pairwise comparisons.** | | | | | | | | |
| --- | --- | --- | --- | --- | --- | --- | --- | --- |
| **Scenario** | **Vendor** | **Scanner model** | **Scan option** | **effective mAs** | **Convolution kernel** | **Body filter** | **Pixel spacing** | **Slice thickness** |
| **CCR-001 VS CCR-002** |  |  |  |  |  |  |  |  |
| **CCR-001 VS CCR-003** |  |  |  |  |  |  |  |  |
| **CCR-001 VS CCR-004** |  |  |  |  |  |  |  |  |
| **CCR-001 VS CCR-005** |  |  |  |  |  |  |  |  |
| **CCR-001 VS CCR-006** |  |  |  |  |  |  |  |  |
| **CCR-001 VS CCR-007** |  |  |  |  |  |  |  |  |
| **CCR-001 VS CCR-008** |  |  |  |  |  |  |  |  |
| **CCR-001 VS CCR-009** |  |  |  |  |  |  |  |  |
| **CCR-001 VS CCR-010** |  |  |  |  |  |  |  |  |
| **CCR-001 VS CCR-011** |  |  |  |  |  |  |  |  |
| **CCR-001 VS CCR-012** |  |  |  |  |  |  |  |  |
| **CCR-001 VS CCR-013** |  |  |  |  |  |  |  |  |
|  |  |  |  |  |  |  |  |  |
| **CCR-002 VS CCR-003** |  |  |  |  |  |  |  |  |
| **CCR-002 VS CCR-004** |  |  |  |  |  |  |  |  |
| **CCR-002 VS CCR-005** |  |  |  |  |  |  |  |  |
| **CCR-002 VS CCR-006** |  |  |  |  |  |  |  |  |
| **CCR-002 VS CCR-007** |  |  |  |  |  |  |  |  |
| **CCR-002 VS CCR-008** |  |  |  |  |  |  |  |  |
| **CCR-002 VS CCR-009** |  |  |  |  |  |  |  |  |
| **CCR-002 VS CCR-010** |  |  |  |  |  |  |  |  |
| **CCR-002 VS CCR-011** |  |  |  |  |  |  |  |  |
| **CCR-002 VS CCR-012** |  |  |  |  |  |  |  |  |
| **CCR-002 VS CCR-013** |  |  |  |  |  |  |  |  |
|  |  |  |  |  |  |  |  |  |
| **CCR-003 VS CCR-004** |  |  |  |  |  |  |  |  |
| **CCR-003 VS CCR-005** |  |  |  |  |  |  |  |  |
| **CCR-003 VS CCR-006** |  |  |  |  |  |  |  |  |
| **CCR-003 VS CCR-007** |  |  |  |  |  |  |  |  |
| **CCR-003 VS CCR-008** |  |  |  |  |  |  |  |  |
| **CCR-003 VS CCR-009** |  |  |  |  |  |  |  |  |
| **CCR-003 VS CCR-010** |  |  |  |  |  |  |  |  |
| **CCR-003 VS CCR-011** |  |  |  |  |  |  |  |  |
| **CCR-003 VS CCR-012** |  |  |  |  |  |  |  |  |
| **CCR-003 VS CCR-013** |  |  |  |  |  |  |  |  |
|  |  |  |  |  |  |  |  |  |
| **CCR-004 VS CCR-005** |  |  |  |  |  |  |  |  |
| **CCR-004 VS CCR-006** |  |  |  |  |  |  |  |  |
| **CCR-004 VS CCR-007** |  |  |  |  |  |  |  |  |
| **CCR-004 VS CCR-008** |  |  |  |  |  |  |  |  |
| **CCR-004 VS CCR-009** |  |  |  |  |  |  |  |  |
| **CCR-004 VS CCR-010** |  |  |  |  |  |  |  |  |
| **CCR-004 VS CCR-011** |  |  |  |  |  |  |  |  |
| **CCR-004 VS CCR-012** |  |  |  |  |  |  |  |  |
| **CCR-004 VS CCR-013** |  |  |  |  |  |  |  |  |
|  |  |  |  |  |  |  |  |  |
| **CCR-005 VS CCR-006** |  |  |  |  |  |  |  |  |
| **CCR-005 VS CCR-007** |  |  |  |  |  |  |  |  |
| **CCR-005 VS CCR-008** |  |  |  |  |  |  |  |  |
| **CCR-005 VS CCR-009** |  |  |  |  |  |  |  |  |
| **CCR-005 VS CCR-010** |  |  |  |  |  |  |  |  |
| **CCR-005 VS CCR-011** |  |  |  |  |  |  |  |  |
| **CCR-005 VS CCR-012** |  |  |  |  |  |  |  |  |
| **CCR-005 VS CCR-013** |  |  |  |  |  |  |  |  |
|  |  |  |  |  |  |  |  |  |
| **CCR-006 VS CCR-007** |  |  |  |  |  |  |  |  |
| **CCR-006 VS CCR-008** |  |  |  |  |  |  |  |  |
| **CCR-006 VS CCR-009** |  |  |  |  |  |  |  |  |
| **CCR-006 VS CCR-010** |  |  |  |  |  |  |  |  |
| **CCR-006 VS CCR-011** |  |  |  |  |  |  |  |  |
| **CCR-006 VS CCR-012** |  |  |  |  |  |  |  |  |
| **CCR-006 VS CCR-013** |  |  |  |  |  |  |  |  |
|  |  |  |  |  |  |  |  |  |
| **CCR-007 VS CCR-008** |  |  |  |  |  |  |  |  |
| **CCR-007 VS CCR-009** |  |  |  |  |  |  |  |  |
| **CCR-007 VS CCR-010** |  |  |  |  |  |  |  |  |
| **CCR-007 VS CCR-011** |  |  |  |  |  |  |  |  |
| **CCR-007 VS CCR-012** |  |  |  |  |  |  |  |  |
| **CCR-007 VS CCR-013** |  |  |  |  |  |  |  |  |
|  |  |  |  |  |  |  |  |  |
| **CCR-008 VS CCR-009** |  |  |  |  |  |  |  |  |
| **CCR-008 VS CCR-010** |  |  |  |  |  |  |  |  |
| **CCR-008 VS CCR-011** |  |  |  |  |  |  |  |  |
| **CCR-008 VS CCR-012** |  |  |  |  |  |  |  |  |
| **CCR-008 VS CCR-013** |  |  |  |  |  |  |  |  |
|  |  |  |  |  |  |  |  |  |
| **CCR-009 VS CCR-010** |  |  |  |  |  |  |  |  |
| **CCR-009 VS CCR-011** |  |  |  |  |  |  |  |  |
| **CCR-009 VS CCR-012** |  |  |  |  |  |  |  |  |
| **CCR-009 VS CCR-013** |  |  |  |  |  |  |  |  |
|  |  |  |  |  |  |  |  |  |
| **CCR-010 VS CCR-011** |  |  |  |  |  |  |  |  |
| **CCR-010 VS CCR-012** |  |  |  |  |  |  |  |  |
| **CCR-010 VS CCR-013** |  |  |  |  |  |  |  |  |
|  |  |  |  |  |  |  |  |  |
| **CCR-011 VS CCR-012** |  |  |  |  |  |  |  |  |
| **CCR-011 VS CCR-013** |  |  |  |  |  |  |  |  |
|  |  |  |  |  |  |  |  |  |
| **CCR-012 VS CCR-013** |  |  |  |  |  |  |  |  |
|  | | | | | | | | |
| Disagree Agree | | | | | | | | |
